# Facile Patterning of Thermoplastic Elastomers and Robust Bonding to Glass and Thermoplastics for Microfluidic Cell Culture and Organ-on-Chip

**DOI:** 10.3390/mi12050575

**Published:** 2021-05-18

**Authors:** Stefan Schneider, Eduardo J. S. Brás, Oliver Schneider, Katharina Schlünder, Peter Loskill

**Affiliations:** 1 Fraunhofer Institute for Interfacial Engineering and Biotechnology IGB, 70569 Stuttgart, Germany; stefan.schneider@igb.fraunhofer.de (S.S.); oliver.schneider@igb.fraunhofer.de (O.S.); 2 NMI Natural and Medical Sciences Institute at the University of Tübingen, 72770 Reutlingen, Germany; Eduardo.Bras@nmi.de (E.J.S.B.); katharina.schluender@uni-tuebingen.de (K.S.); 3 Department of Biomedical Engineering, Faculty of Medicine, Eberhard Karls University Tübingen, 72076 Tübingen, Germany; 4 3R Center Tübingen for In Vitro Models and Alternatives to Animal Testing, 72076 Tübingen, Germany

**Keywords:** microfluidics, organ-on-chip, thermoplastic elastomer, microfabrication

## Abstract

The emergence and spread of microfluidics over the last decades relied almost exclusively on the elastomer polydimethylsiloxane (PDMS). The main reason for the success of PDMS in the field of microfluidic research is its suitability for rapid prototyping and simple bonding methods. PDMS allows for precise microstructuring by replica molding and bonding to different substrates through various established strategies. However, large-scale production and commercialization efforts are hindered by the low scalability of PDMS-based chip fabrication and high material costs. Furthermore, fundamental limitations of PDMS, such as small molecule absorption and high water evaporation, have resulted in a shift toward PDMS-free systems. Thermoplastic elastomers (TPE) are a promising alternative, combining properties from both thermoplastic materials and elastomers. Here, we present a rapid and scalable fabrication method for microfluidic systems based on a polycarbonate (PC) and TPE hybrid material. Microstructured PC/TPE-hybrid modules are generated by hot embossing precise features into the TPE while simultaneously fusing the flexible TPE to a rigid thermoplastic layer through thermal fusion bonding. Compared to TPE alone, the resulting, more rigid composite material improves device handling while maintaining the key advantages of TPE. In a fast and simple process, the PC/TPE-hybrid can be bonded to several types of thermoplastics as well as glass substrates. The resulting bond strength withstands at least 7.5 bar of applied pressure, even after seven days of exposure to a high-temperature and humid environment, which makes the PC/TPE-hybrid suitable for most microfluidic applications. Furthermore, we demonstrate that the PC/TPE-hybrid features low absorption of small molecules while being biocompatible, making it a suitable material for microfluidic biotechnological applications.

## 1. Introduction

Microfluidics technology provides powerful tools for a wide range of applications in the area of biotechnology and biochemistry. The combination of low reagent requirements (nL to low mL) and small footprints (mm to low cm) make microfluidic platforms very attractive alternatives to traditional bench-top methods. Two prime examples are portable diagnostic tools [1] and organ-on-chip (OoC) models [2]. The microscale dimensions of microfluidic systems allow for precise control of reaction conditions, low reagent usage, and portability, which are key advantages for the utilization as point-of-need detection systems [3,4]. Additionally, microfluidics technology enables the construction of elaborate networks of fluidic channels and chambers with tailored dimensions and geometries. This allows for the creation of defined microphysiological environments on the scale of individual cells and tissues, making microfluidics an attractive platform for cell culture and tissue engineering. An important example of this is the OoC technology, which has emerged over the last decade as an alternative to animal models for biomedical and pharmaceutical research, constituting powerful tools for disease modeling and drug development [5].

The most common material used for the fabrication of microfluidic chips is the elastomer polydimethylsiloxane (PDMS), which is mainly due to its optical transparency [6], gas permeability [7], biocompatibility, and simple fabrication methods. PDMS allows for patterning by replica molding of microstructured master molds and facile bonding to other PDMS modules or glass substrates by oxygen plasma activation [8]. Moreover, bonding to different types of thermoplastics can be achieved utilizing chemical surface functionalization [9,10,11,12]. However, the limitations of PDMS are its porosity and hydrophobic nature, leading to the uncontrolled absorption of small hydrophobic molecules [13] and evaporation of water, inducing vulnerability to osmolality shifts [14]. Further disadvantages are limited mechanical stability as well as the leaching of uncured oligomers from bulk PDMS into the patterned channels [15], potentially affecting e.g., biological experiments [16]. Furthermore, compared to thermoplastics, PDMS-based systems suffer from low fabrication throughput and high material cost, hindering large-scale production and the commercialization of PDMS-based systems in general [17].

With these shortcomings in mind, several attempts toward PDMS-free microfluidic platforms have been made over the last years, with thermoplastics being the most frequently applied alternative material. However, bonding of thermoplastic materials is more challenging than PDMS. Thermal fusion, solvent, or adhesive bonding are frequently applied strategies for building thermoplastic microfluidic systems. However, all of these approaches possess several limitations:

- Thermal fusion bonding requires contact pressure and temperatures in the range of the glass transition temperature of the respective material. These working conditions are necessary for polymer chain fusion at the interface of the two substrates. The resulting bond is strongly dependent on well-optimized parameters for heat and pressure in order to achieve sufficient bonding strength without channel deformation [18].
- Solvent bonding is based on surface treatment with a suitable solvent to increase the mobility of polymer chains at the interface of the polymer substrates, resulting in a stable weld after complete evaporation [19]. However, excessive solvent exposure poses the risk of channel destruction and solvent residuals, which can be problematic for biological applications.
- Similarly, adhesive bonding can result in channel clogging due to excessive spreading of adhesives into microstructures and often needs to be addressed by optimized glue deposition e.g., by adhesive contact printing [20]. Furthermore, depending on the desired application, adhesives need to be chosen carefully with respect to bonding strength, material compatibility, absorption properties, and cytotoxicity [21].

More recently, thermoplastic elastomers (TPE) have gained traction for the fabrication of microfluidic modules [22]. A commonly applied subtype of TPE is styrenic block copolymer, such as styrene–ethylene/butylene–styrene (SEBS). They consist of a thermoplastic polystyrene block attached to both ends of the elastomeric middle segment [23]. According to their chemical structure, these materials share thermoplastic and elastic properties and feature two glass transition temperatures for both elastic and thermoplastic segments. Glass transition occurs typically at approximately −65 °C and 90 °C for the soft ethylene/butylene block and the rigid styrene block, respectively [22]. Above the glass transition temperature of the rigid styrene phase, SEBS loses its elasticity and starts to melt. SEBS is compatible with several melt processing techniques that are commonly used for thermoplastics such as hot embossing [24] and injection molding [25]. Thereby, SEBS is structured above the glass transition temperature of the rigid styrene phase. After cooling, the initial elastic character is regained, and mechanically flexible molds with defined features are obtained. Furthermore, SEBS can be dissolved in a suitable solvent and used for replica molding similarly to elastomeric materials such as PDMS [26]. Compared to standard thermoplastics, an important advantage of SEBS is its self-adhesive surface, enabling a relatively simple bonding to itself or a variety of other surfaces. When compared to PDMS, the material cost of SEBS is significantly lower, with a price point for the bulk material being below 20 €/kg, while the typical cost of PDMS is close to 100 €/kg. Additionally, SEBS benefits from a reduced absorption of small hydrophobic molecules compared to PDMS [25,26].

Despite all of the apparent advantages of TPE, its usage in the field of microfluidics is still not widespread. One of the challenges of working with TPE-based devices is that they tend to have tedious handling procedures. Stacking and aligning multiple, thin layers of TPE is difficult due to a relatively low Young’s modulus [22,25,27] and instantaneous adherence of the material to several surfaces. Moreover, although it features self-adhesive properties, for some materials, further functionalization is required to ensure long-term bonding.

Here, we present a solution to counteract the difficulty in fabrication and handling of TPE-based systems by utilizing a composite material combining TPE and polycarbonate (PC), to which we will refer to as a PC/TPE-hybrid. This novel material combines the easy patterning and bonding capabilities of TPE and allows for facile handling and optimized optical properties through the reinforcement of TPE with a transparent, thermoplastic film. We provide a thorough characterization of this hybrid material from an application standpoint in terms of both relevant physical and (bio)chemical properties. Furthermore, we also present an easy to implement surface functionalization approach, based on silane chemistry, for long-term and high-pressure bonding of TPE to glass substrates.

## 2. Materials and Methods

### 2.1. SU-8 Microstructures

Microstructures on silicon wafers were fabricated using standard SU-8 microfabrication [28]. Briefly, SU-8 2075 (Kayaku Advanced Materials, Inc., Berlin, Germany) was spin coated onto a 150 mm silicon wafer (Siegert Wafer, Aachen, Germany) at 1750 rpm final speed for 34 s and soft baked for 5 min at 65 °C, subsequently heated up to 95 °C, and baked for 15 min. Microstructures were designed using CorelCAD software, and the required photomask was purchased from an external provider (KOPP-desktopmedia, Nufringen, Germany). The SU-8 layer was patterned by exposing the sample through the photomask to ultraviolet (UV) light (ABM Series 60 Exposure Systems; ABM, Inc., New York City, NY, USA), at 21 mW/cm² for 5 s. The post exposure bake was performed at 65 °C for 5 min followed by a similar temperature ramp as the pre-exposure bake and then baked for an additional 9 min at 95 °C. The structures were developed in propylene glycol methyl ether acetate (Sigma-Aldrich, St. Louis, MI, USA) for 12 min and hard baked at 150 °C for 30 min.

### 2.2. PDMS Mold Fabrication

To cast PDMS onto the silicon wafer, a fluoroelastomer-based O-ring (138 mm × 2 mm FPM 75, Dichtelemente arcus, Seevetal Germany) was placed concentrically on the wafer. Both, wafer and O-ring, were clamped between two 5 mm thick polymethyl methacrylate (PMMA) plates (Oroglas cast acrylic glass, Trinseo, Berwyn, PA, USA) with the upper one featuring two ports for connecting 50 mL syringes (BD Plastipak, Becton Dickinson, Heidelberg, Germany). PDMS (Sylgard 184, Dow Corning, Wiesbaden, Germany) was mixed in a 10:1 (base:curing agent) mass ratio, degassed in a desiccator, and filled into one of the syringes connected to the PMMA plate. By slowly pulling the plunger of the other syringe, PDMS was injected into the O-ring sealed cavity on the wafer. PDMS was cured overnight at 60 °C (Universal Oven UN110, Memmert, Büchenbach, Germany). After curing, the PMMA plates were removed, and the PDMS was peeled off the wafer and cleaned with isopropanol as well as compressed nitrogen.

### 2.3. Epoxy Master Mold Fabrication

To fabricate the epoxy mold, a custom-built, aluminum molding tool was designed (Supplementary Information Figure S1). The tool was manufactured by an external service provider (CNCTeile24, Berlin, Germany) and consists of a base plate featuring a vacuum chuck for fixation of the PDMS mold by contact vacuum and a hose connection for a vacuum pump (LABOPORT N 86 KN.18, KNF, Hamburg, Germany). The middle part of the tool, an aluminum ring (8 mm height, 110 mm inner diameter), is placed on top of the PDMS negative mold and forms a cavity for the epoxy resin. This cavity is closed with a PMMA plate sealed with an O-ring (125 mm × 2 mm FPM 75, Dichtelemente arcus, Seevetal, Germany) and connected to the tool by screws. Identically to the PDMS molding setup, the PMMA plate features ports for the connection of syringes.

A two-component (parts “A” and “B”), high-temperature stable epoxy resin (EpoxAcast 670 HT, Smooth-On, Macungie, PA, USA) was mixed with an epoxy thinner (Epic Epoxy Thinner, Smooth-on, Macungie, PA, USA) for reduced viscosity of the freshly prepared epoxy mixture (part A:thinner:part B = 100:10:17.6 mass ratio). Air bubbles in the mixture were removed by placing it in a desiccator at reduced pressure for 3 min followed by 10 min in an ultrasound bath (JP-031S, RS PRO, Frankfurt, Germany).

Subsequently, the epoxy resin was filled into the cavity of the molding tool in the same way as described for PDMS molding. For curing, the tool was left at room temperature for 24 h, while the pump for contact pressure was constantly running. Subsequently, the tool was disconnected from the pump and placed in an oven (Universal Oven UN110, Memmert, Büchenbach, Germany) at 60 °C for another 24 h. Afterwards, the epoxy master was removed from the tool and tempered for 2 h at 80 °C followed by 3 h at 150 °C in an oven (Universal Oven UN30, Memmert, Büchenbach, Germany) and slowly cooled down to room temperature.

### 2.4. Hot Embossing

Commercial TPE pellets (Mediprene OF400M, HEXPOL TPE AB, Åmål, Sweden) were extruded to an approximately 750 µm thick foil by an external service provider (Fraunhofer Institute for Process Engineering and Packaging IVV, Freising, Germany). For standard TPE hot embossing, TPE slaps of 11 × 11 cm² were laminated on a 250 µm thick polytetrafluoroethylene (PTFE) foil (High-tech-flon films and fabrics, Konstanz, Germany) as temporary carrier using a hand held pressure roller (Steinel, Herzebrock-Clarholz, Germany). Subsequently, the epoxy mold was placed on top of the TPE sheet, and the assembly was transferred onto a 150 mm silicon wafer (Siegert Wafer, Aachen, Germany). For PC/TPE-hybrid hot embossing, the TPE slabs were laminated onto a 500 µm thick PC foil (Makrofol DE 1-1, Covestro, Leverkusen, Germany). After placing the epoxy on top of the TPE, the PC foil was placed on a 150 mm mirror polished stainless steel plate (TGA GmbH, Leun, Germany) spray coated with a release agent (Weicon, Münster, Germany). 

In both cases, the respective assembly was then transferred into a hot press (LabManual 300, Fontijne Presses, Delft, Netherlands) preheated to 130 °C. The epoxy stamp was pressed into the TPE at 130 °C maintaining a pressure of 0.8 MPa for 10 min and subsequently cooled down below 40 °C before opening the press. After hot embossing, the TPE was peeled off from the epoxy using a few drops of isopropanol and then dried using compressed nitrogen.

### 2.5. TPE Bonding 

PC/TPE-hybrid layers were bonded to the following substrates: (i) TPE (unprocessed 750 µm extruded sheet); (ii) cyclic olefin copolymer (COC, 240 µm EUROPLEX Film 0F305, Röhm, Darmstadt, Germany); (iii) PC (175 µm Makrofol DE 1-1, Covestro, Leverkusen, Germany); (iv) polystyrene (PS, cell culture dish, CELLSTAR, Greiner Bio-One, Frickenhausen, Germany); (v) glass (Microscope slide, soda-lime glass, Thermo scientific, Waltham, MA, USA).

In order to bond the PC/TPE-hybrid layer to the different substrate combinations, three different pre-treatments were applied: (i) Samples referred to as “non-activated” did not undergo any additional surface treatment. The “activated” samples underwent (ii) surface oxidation via oxygen plasma or (iii) surface functionalization using Bis-[3-(trimethoxysilyl)-propyl]-amine (Bisamino Silane). For the oxygen plasma activation, the PC/TPE-hybrid material was exposed to a 50 W, 100% oxygen plasma (Zepto, Diener, Ebhausen, Germany) for 60 s before lamination onto the respective substrate. The silane functionalization was only utilized to bond PC/TPE-hybrid layers to glass substrates. First, the PC/TPE-hybrid was exposed to an oxygen plasma (60 s, 50 W; Zepto, Diener, Ebhausen, Germany) and subsequently incubated for 20 min at 80 °C in a bisamino silane (Sigma-Aldrich, St. Louis, MI, USA) solution (2% (v/v) bisamino silane; 1% (v/v) Milli Q water in technical grade isopropanol). Then, the treated PC/TPE-hybrid material was rinsed three times with technical grade isopropanol, which was followed by three rinsing steps with a 70% (v/v) ethanol solution. After washing, the samples were incubated for 20 min in a 70% (v/v) ethanol solution at room temperature, followed by a drying step using compressed nitrogen. Meanwhile, the glass slide was thoroughly cleaned by immersing the sample in a non-ionic detergent (Alconox ®, Sigma-Aldrich, St. Louis, MI, USA) solution (1% (w/w) in DI water) at 65 °C for 30 min, followed by multiple rinsing steps using DI water. After drying using compressed nitrogen, the glass surface was activated via oxygen plasma (60 s, 50 W; Zepto, Diener, Ebhausen, Germany). 

In all cases, PC/TPE-hybrid layers were bonded onto the respective substrates with the assistance of a hand held pressure roller (Steinel, Herzebrock-Clarholz, Germany). This was to ensure equal pressure distribution and also to assist in the removal of any small air pockets that otherwise could have been trapped between the two layers. Subsequently, the combined devices were placed overnight in an oven (Universal Oven UN110, Memmert, Büchenbach, Germany) set to 60 °C.

### 2.6. Optical Characterization

The UV-VIS absorption spectroscopy was conducted using a plate reader (Infinite M200 Pro, Tecan, Männedorf, Switzerland). Samples were placed in the bottom of PS based 6-well plates (CELLSTAR, Greiner Bio-One, Frickenhausen, Germany) and light absorption at wavelength between 300 nm and 600 nm was analyzed. The resulting values were normalized to an empty well on the plate to account for the impact of the plate material itself. In the case of the Ostemer Crystal Clear (Ostemer ® 322 Crystal Clear, Ostemers, Stockholm, Sweden) substrate, a small disc was prepared in accordance to the manufacturer’s instructions. Briefly, both parts of the commercial kit were mixed at a ratio of 1.09:1 (v/v) and subsequently degassed for 10 min in a desiccator. The degassed mixture was initially cross-linked using a UV exposure unit (9 s, 125 mW/cm^2^, λ = 365 nm LED UV mini-Oven, Novachem, Dunboyne, Ireland). Then, the substrate was baked at 110 °C for 2 h.

### 2.7. Maximum Working Pressure Testing

To test the bond strength between the PC/TPE-hybrid and the substrates of choice, channel inlets were equipped with luer connectors (BDMFTLL-9, Nordson MEDICAL, West Lake, OH, USA) and channel outlets sealed using epoxy glue (UHU PLUS ENDFEST, UHU, Bühl, Germany). After completely curing the epoxy glue for 24 h at room temperature, samples were split into two groups: half of the samples were tested immediately (Day 0 samples), the rest were submersed in a reservoir containing PBS (Dulbecco’s phosphate-buffered saline w/o calcium w/o magnesium, Biowest, Nuaillé, France) and placed in an incubator (Heraeus BBD 6220, Thermo Scientific, Waltham, MA, USA) for 7 days (Day 7 samples) at 37 °C, 95% humidity, and 5% CO_2_. In order to measure the maximum working pressure enabled by each bonding method, a gas line providing high-pressure (Max = 7.5 bar) nitrogen was connected to the microfluidic chip via luer connectors. Then, the connected chip was placed under water in a basin to facilitate the detection of gas leakage from the chip (Supplementary Information Figure S2). The integrity of the microfluidic chip was observed, while the pressure was increased in increments of 0.5 bar every 10 s until some form of failure, either by material deformation or channel delamination, was registered. 

### 2.8. Absorption Studies

TPE chips featuring a straight channel (1.2 mm length, 400 µm width, 100 µm height) were fabricated via bisamino silane-assisted PC/TPE-glass bonding. To connect pipette tips to the chips, PMMA connectors were fabricated by laser cutting (VLS2.30, Universal Laser Systems, Scottsdale, AR, USA) and bonded to the PC layer using biocompatible, double-sided adhesive tape (ARcare 90106, Adhesives Research, Limerick, Ireland). PDMS chips featuring the same structures were fabricated by replica molding. PDMS was cured for 4 h at 60 °C, peeled off the wafer, and cut to chip size. Access ports were punched with a biopsy puncher (504529, World Precision Instruments, Sarasota, FI, USA). The channel side of the PDMS slab was thoroughly washed with isopropanol and blow-dried with nitrogen; then, residual particles were removed with scotch tape. Microscope slides were washed with acetone and isopropanol and then subsequently blow-dried with nitrogen. PDMS and glass substrate were treated with oxygen plasma (30 s, 50 W; Zepto, Diener, Ebhausen, Germany ), and the PDMS modules were placed with the channel side facing downwards onto the glass. The chips were left overnight at 60 °C to ensure proper bonding. 

To investigate the partitioning of small molecules into the chip material, three different rhodamine dyes were utilized (Rhodamine B, 83689; Rhodamine 6G, 83697; Rhodamine 101, 83694; all Sigma-Aldrich, St. Louis, MI, USA). All dyes were reconstituted in PBS to a final concentration of 100 µM. Each solution was filtered through a 0.22 µm pore size filter (P666.1, Carl Roth, Karlsruhe, Germany). For each chip material and rhodamine, three different chips were analyzed. 

Prior to introducing dye solutions into the channels, chips were treated with oxygen plasma (5 min, 50 W; Zepto, Diener, Ebhausen, Germany ), reproducing conditions deployed in the cell culture experiment. An empty pipet tip (300 µL, SAPPHIRE PIPETTE TIP, Greiner Bio-One, Frickenhausen, Germany) was added to the outlet port; then, a pipet tip containing 50 µL of respective rhodamine solution was added to the inlet. The channel was flushed by applying pressure to the inlet tip via the hand held pipet until both liquid columns of the inlet and outlet tips were equilibrated. After filling, all samples were spatially fixed in 3D-printed holders designed to fit into the microscope stage. Samples were imaged utilizing a fluorescence microscope (Leica DMi8, Leica Microsystems) with a magnification of 10x and a rhodamine filter set (λ_Excit._ = 546/10 nm, λ_DM_ = 560 nm, λ_Emiss._ = 585/40 nm). Images were taken in the center of the channel and focused in the z-direction to the TPE/PDMS–glass interface. Imaging conditions were kept the same for each rhodamine between materials and imaging time points. Starting immediately after rhodamine injection, samples were imaged every 24 h for three days. All samples were imaged before and after washout of the rhodamine solutions at *t* = 72 h. In between each recording, samples were stored in an enclosed light-protected box containing a water-filled crystallization dish to prevent excessive evaporation of the different rhodamine solutions from the pipet tips. After 72 h, the dye solution was washed out of the chip by manually flushing the channel three times with approximately 80 µL of PBS via pipet tips. 

Fluorescence images were processed by correcting small sample tilts, centering the channel, and cropping a defined region around the channel center via a custom Python script (Supplementary Information; https://github.com/loslab/rhodabs, accessed on 17th of May 2021). Fluorescence intensity profiles perpendicular to the channels were extracted by taking the mean of the fluorescence values along the full, visible channel length in each tilted and cropped image. 

For each rhodamine and material combination, profiles were further averaged among all replicates (as leakage occurred after 48 h in the PDMS–rhodamine 6G sample 2; this sample was excluded from the mean value of time points 72 h and 72 h washout). Fluorescence intensity was normalized to the mean value in the channel center of all samples filled with the same rhodamine solution at *t* = 0 h. Displayed fluorescence images are cropped, showing 50% of the full, analyzed raw image. 

### 2.9. Cell Culture Experiments

For on-chip cell culture experiments, commercially available cryopreserved human umbilical vein endothelial cells (HUVEC) from pooled donors (C2519A, Lonza, Basel, Switzerland) were cultured in endothelial cell growth medium (EGM-2 BulletKit, CC-3162, Lonza) and expanded before chip seeding in 75 cm² filter cap cell culture flasks (CELLSTAR, Greiner Bio-One, Frickenhausen, Germany). For chip seeding, adherent cells were washed with PBS and dissociated by 3–5 min incubation using 0.05% (v/v) trypsin (Trypsin-EDTA Solution 10X, SIGMA Life Science, St. Louis, MI, USA) in Versene solution (Versene 1:5,000 1X, Gibco, Waltham, MA, USA). After trypsin inactivation by adding equal amounts of cell medium, cells were centrifuged (Multifuge 3S-R, Heraeus, Waltham, MA, USA) at 1000 rpm (216 g) and re-suspended in cell medium to a final cell concentration of 6 × 10^6^ cells/mL.

Cell culture experiments on-chip were performed in PC/TPE-glass chips, identical to the ones from the absorption studies. Chips were sterilized by oxygen plasma for 5 min and subsequently flushed with PBS. The channel surfaces were coated by filling 0.1 mg/mL collagen-I (FibriCol, Catalog #5133, Advanced BioMatrix, Carlsbad, CA, USA) in PBS into the channel. After incubation for 1 h under culture conditions (37 °C, 95% humidity, 5% CO_2_), chips were flushed with PBS. 

For cell seeding, a pipette tip containing 30 µL of cell suspension was attached to the channel inlet and the cells were injected into the channel. Subsequently, chips were flipped into an upside-down position for 15 min and then flipped back to the upright position to achieve cell attachment on top and bottom of the channel. After 1 h, pipette tips were topped up, and cells were cultured under static culture conditions using media-filled pipet tips over a period of 3 days. Media were changed daily.

### 2.10. Live/Dead Staining

After 3 days of culture, a live/dead staining was performed. Fluorescein diacetate (FDA) (F7378, Sigma-Aldrich, St. Louis, MI, USA) for labeling viable cells, propidium iodide (PI) (P4170, Sigma-Aldrich, St. Louis, MI, USA) for labeling the nuclei of dead cells, and Hoechst 33342 (62249, Thermo Scientific, Waltham, MA, USA) for labeling the nuclei of all (viable and dead) cells were used. All dyes were diluted in cell culture medium without fetal bovine serum, and incubations were performed under culture conditions.

For each chip, medium containing 1 µg/mL of Hoechst was flushed into the channel and incubated for 20 min. Subsequently, chips were flushed by gravity flow with medium containing 8 µg/mL of FDA and 20 µg/mL of PI and incubated for 5 min. Chips were washed with PBS by gravity flow and imaged immediately. Images of stained HUVECs on the top and bottom of each channel were acquired at 37 °C using a fluorescence microscope with heated enclosure (Leica DMi8, Leica Microsystems, Wetzlar, Germany).

## 3. Results and Discussion

### 3.1. Patterning Process and Characterization

**Figure 1:**
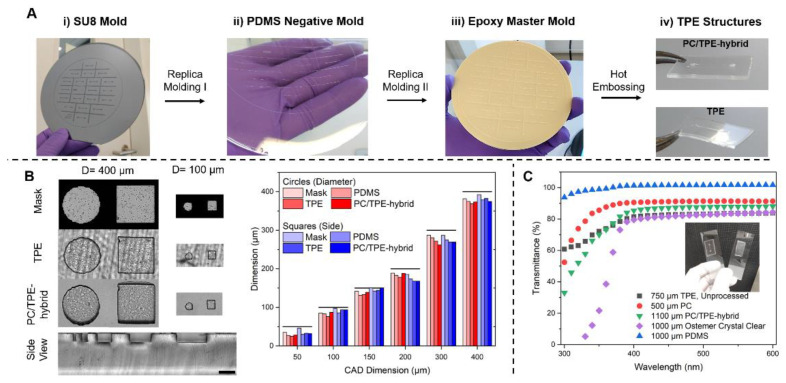
(**A**) TPE microfabrication process: (i) A silicon wafer with SU-8 patterns serves as a master for (ii) the negative PDMS mold, which is replicated once more in the form of (iii) the epoxy master mold. Hot embossing using the epoxy mold enables fabrication of (iv) TPE and PC/TPE-hybrid-based microfluidic platforms. (**B**) Micrographs of features fabricated by hot embossing into TPE and PC/TPE-hybrid as well as the used photomask. A close-up side view shows microstructures in the TPE part of a PC/TPE-hybrid. The full-sized side view image of the PC/TPE-hybrid, additionally showing the transition from TPE to PC, is presented in the supplementary information. Scale bar represents 200 µm. Comparison of feature dimensions between different structures, materials, and the original CAD design. The features have a height of approximately 100 µm. (**C**) UV-VIS transmittance spectra of TPE, PC, and PC/TPE-hybrid as well as of an Ostemer Crystal clear disc and a PDMS slab for comparison purposes. The inset comprises a photograph of a PC/TPE-hybrid device (left) and a TPE device (right) demonstrating the difference in transparency.

As the main process for the fabrication of TPE-based devices, we established a tailored hot embossing approach (Figure 1A): Initially, SU-8 patterns on silicon wafers are created via traditional UV-lithography. By casting PDMS on the patterned wafer, a negative mold is generated that serves as basis for the fabrication of an epoxy-based master mold capable of repeated use. The epoxy master mold can be used to pattern both the TPE layer and the PC/TPE hybrid material. The PC/TPE-hybrid is a composite material obtained by thermally fusing a thin sheet (500 µm) of PC to the TPE substrate (750 µm), resulting in a final thickness of ≈1100 µm. In our fabrication approach, the thermal fusion process is performed simultaneously with the patterning during the embossing step, resulting in a process time of approximately 45 min (30 min press preheating, 10 min embossing, and 5 min cool down), which is significantly shorter than typical PDMS baking times (>1.5 h). Compared to the original thin, flexible TPE, the PC/TPE-hybrid is a stiffer material capable of sustaining its own weight. Thereby, aligning and stacking during chip fabrication is significantly improved, and the risk of folding onto itself, as well as the likelihood of unwanted adhesion to another surface, is reduced (Figure 1A iv).

The PC/TPE-hybrid presents additional application benefits: In the case of the standalone TPE device, the surface is quite rough because of the temporary carrier during hot embossing. This leads to a non-uniform background, which is a hindrance for bright-field microscopy. However, the PC/TPE-hybrid presents a more uniform background appearance, as the TPE is bonded onto a polished surface provided by the transparent PC foil (Figure 1B). The hot embossing process leaves an accurate channel profile with little to no warping of the TPE part as seen in the side view of a PC/TPE-hybrid layer (Figure 1B and Supplementary Information Figure S3). 

The PC/TPE-hybrid approach also allows for a slightly higher feature precision (Figure 1B). Compared to the photomask dimensions, a 94.3 ± 2.5% (mean ± standard deviation) precision in TPE and 96.5 ± 3.9% precision in PC/TPE-hybrids is achieved in the 400 µm to 100 µm feature range. In the case of the 50 µm feature size and below, both fabrication approaches are limited and fall into a ≈70% precision range. Despite this, the demonstrated approach is well suited for replicating structures with feature sizes above 50 µm and aspect ratios ≤1, reflecting the typical dimension range of current OoC systems [29,30].

Since many microfluidic applications rely on microscopy or the use of integrated optical sensors for experimental read-outs [31,32], the optical properties of the PC/TPE-hybrid were analyzed. For comparison, common substrates used for microfluidic applications such as PDMS and Ostemer Crystal Clear were included [33,34]. Additionally, the individual components of the PC/TPE-hybrid, the unprocessed, extruded TPE, and the 500 µm PC foil were analyzed (Figure 1C). The composite of PC and TPE has an improved optical performance compared to the raw TPE material and Ostemer Crystal Clear. This is probably mainly due to the decrease in surface roughness, reducing light scattering, which was obtained with the addition of the transparent PC sheet. Therefore, adding a cheap, transparent thermoplastic foil to TPE provides a simple method of achieving a highly transparent composite material. While PDMS reveals a nearly constant transmission over the full, tested spectra, all other investigated materials display a drop in transmission below 400 nm. 

### 3.2. Device Bonding Strategies

**Figure 2:**
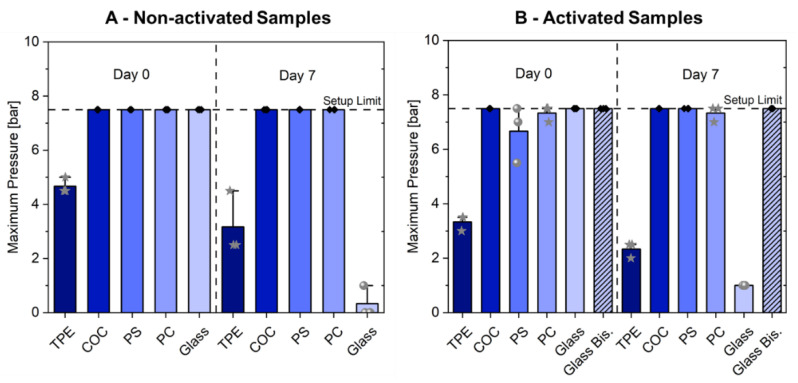
Maximum working pressure (**A**) non-activated samples and (**B**) activated samples bonded to different substrates are able to withstand. Day 0 refers to devices tested right after the overnight thermal bonding. Day 7 refers to devices stored for one week submersed in PBS at 37 °C. The star-labeled data points belong to devices that failed due to material deformation, while spheres belong to devices that resulted in delamination from the substrate. Samples labeled with a black circle reached the maximum pressure of the setup without any form of failure. For each condition, three independent experiments were conducted (only for PS, non-activated, day 0: *N* = 2).

Two approaches for material bonding were tested. The first relied solely on the use of thermal bonding between the PC/TPE-hybrid and the respective substrate, while the second approach relied on some form of activation prior to the thermal bonding step. Samples were activated either via oxygen plasma treatment or via surface functionalization with bisamino silane (Figure 2). 

Some microfluidic applications rely on freshly fabricated devices for one-time use, e.g., point-of-care diagnosis [35], while others utilize devices over longer periods of time in a heated, humid environment, e.g., OoC systems [36]. Hence, all material combinations were tested at two different time-points: after overnight bonding and after 7 days completely submerged in PBS, while being kept at 37 °C in a cell culture incubator.

In case of freshly fabricated devices, simple overnight bonding at 60 °C is sufficient for achieving highly stable bonds between TPE and itself, several thermoplastics, and even glass surfaces (Figure 2A). For all combinations, the bonding is strong enough to withstand typical pressures (< 2 bar) suitable for a wide range of microfluidic applications [37]. In the case of TPE bonded to thermoplastics or glass, the obtained microfluidic chips withstand even the maximum pressure output of the system (7.5 bar). Devices consisting of PC/TPE-hybrid layers and a 750 µm thick layer of extruded TPE could not withstand pressures higher than 4 bar: this pressure resulted in deformation of the TPE bottom substrate. Nevertheless, no delamination at the interface of both layers was observable. 

Prior activation of the TPE surface did not result in increased bonding strength. On the contrary, the activation of TPE and bonding to PS resulted in delamination for all three samples, whereas all non-activated samples could withstand the maximum setup pressure. Borysiak et al. reported improved but reversible room temperature bonding of SEBS to itself or PS when combined with oxygen plasma treatment before lamination. However, similar to our findings, irreversible bonding required elevated temperatures (75 °C) regardless of whether the surfaces were exposed to oxygen plasma or not [26].

After a 1-week submersion in PBS at 37 °C, the devices bonded to thermoplastics still withstood the maximum pressure output, whereby plasma activation did not have any observable effect (Figure 2B). Plasma-assisted bonding to PC failed just below the maximum pressure output but only due to deformation of the 175 µm thin PC foil and not because of delamination. Overall, this indicates that oxygen plasma activation of TPE is not required for long-term stable bonding to thermoplastics.

Devices bonded to glass initially featured strong bond strengths independent of activation status. Yet, after 7 days of submersion, devices bonded both without and with prior plasma activation delaminated at very low applied pressures (≤1 bar). However, the devices bonded after bisamino silane functionalization maintained strong bonding with similar bonding strength as the thermoplastic combinations. The bisamino silane surface functionalization method is based on previous reports of similar surface activations [9]. Activating the TPE surface via oxygen plasma appears to promote the bonding of the Bisamino Silane molecules, leading to the creation of a highly reactive network of silane groups at the surface of the TPE. Through surface activation of the glass substrate via oxygen plasma, hydroxyl groups are created [38], which are then available to react with the silane groups from the activated TPE. This combination, followed by an incubation at 60 °C to promote bond strength, provides a robust bonding between TPE and glass (Figure 2B). This is particularly of importance since the successfully achieved bonding of TPE to glass extends the application range to instrumented microfluidic devices, which often include thin-film sensors built on glass substrates [39].

The maximum pressures achieved for the different material combinations are comparable or even higher than the ones achievable with other reported methods: Lachaux et al. reported 2 bar and 1 bar burst pressures for SEBS–SEBS and SEBS–glass bonds respectively, which were achieved by bonding at 85 °C for 1 h [40]. Borysiak et al. reported maximum pressures of 4.1 bar without failure for bonding of SEBS to itself, PS, or glass surfaces applying 75 °C for 30 min [26]. When compared to PDMS bonded to itself [41], glass [42], or thermoplastics [43,44,45], similar or even higher values of bonding strength can be achieved using our presented TPE-based approach (Table 1), even after 7 days of exposure to buffer.

### 3.3. (Bio)chemical Characterization

#### 3.3.1. Small Molecule Absorption

Preventing the absorption of molecules from the solution into the bulk materials of the chips is of upmost importance due to the high surface-to-volume ratios seen in microfluidic systems, which can lead to the rapid drop of concentrations in the perfused solution [46]. In biosensing assays, target molecule absorption leads to a reduced sensibility of the system, while in cell culture applications, the absorption of specific cellular outputs or injected compounds can affect system read-outs [16,47].

**Figure 3:**
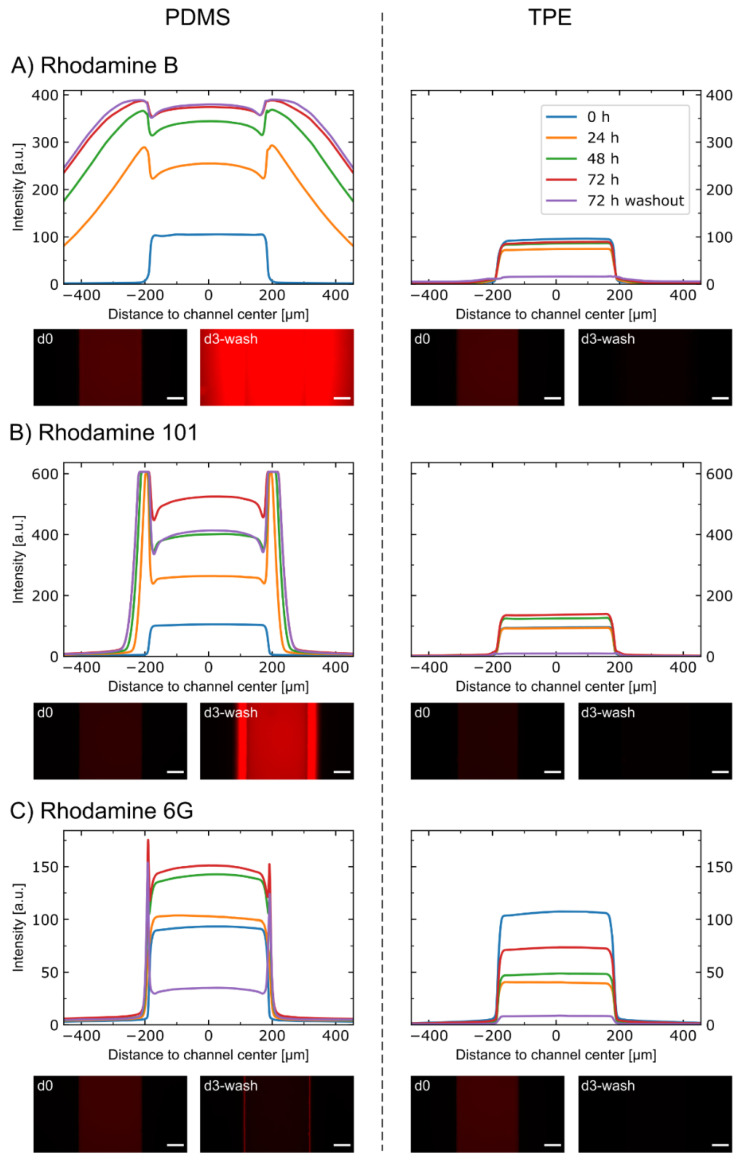
Absorption of rhodamine molecules into PDMS-based and TPE-based microfluidic devices. Average intensity profiles at different time points for chips incubated with (**A**) rhodamine B, (**B**) rhodamine 101, and (**C**) rhodamine 6G solutions. After 72 h of incubation, the solutions were washed out, and the remnant profile was acquired. Micrographs of the channels from day 0 (d0) and from day 3, after washout (d3-wash) are also shown. The scale bars refer to 100 µm. *N* = 2 for PDMS-rhodamine 6G 72 h and 72 h washout. All others are *N* = 3.

To assess the absorption of small molecules into TPE, a comparative study against PDMS was performed. Three different rhodamine dye molecules, with similar molecular weights (479–491 g/mol), were used to account for the effect of different hydrophobicity (2.13 ≤ Log P ≤ 7.8) [48]. Dye solutions were injected into the microchannels, and the fluorescence profiles in the microchannels were monitored at different time points (Figure 3).

In the TPE devices, there is no visible change in the signal profiles for all three tested molecules. In the PDMS devices, a high absorption of both rhodamine B and rhodamine 101 is evident, with the former appearing to have a higher absorption rate than the latter. Similarly, a partitioning of rhodamine 6G into the PDMS can be observed, yet to a much lower extent than for the other two dyes. The absorption of the three dyes is demonstrated not only by the increasing width of the profile over time but also by the remaining high intensity after washout. The increase in intensity over time in the main channel in the PDMS devices indicates an absorption into the PDMS also above the channel. However, the total intensity on different days can be affected by a variety of factors such as absorption to the tubing and connectors, bleaching of fluorescence dye, variation in lamp intensity, and evaporation. Hence, we focused on the lateral profiles of the fluorescent signal and effect of washout for interpretation of the results. 

Our observation is in line with other studies using SEBS formulations without oil additives for microfluidic chip fabrication: Domansky et al. could show significantly lower absorption of rhodamine B and the drug pirfenidone in so-called oil-free SEBS compared to PDMS [25]. In general, low absorption rates of small molecules make TPE an attractive alternative for microfluidic cell culture platforms. The ability to precisely deliver cellular stimulants while not losing cellular outputs provides TPE with an advantage over common materials used in the field, such as PDMS [13,47,49].

#### 3.3.2. Applicability for Cell Culture

**Figure 4:**
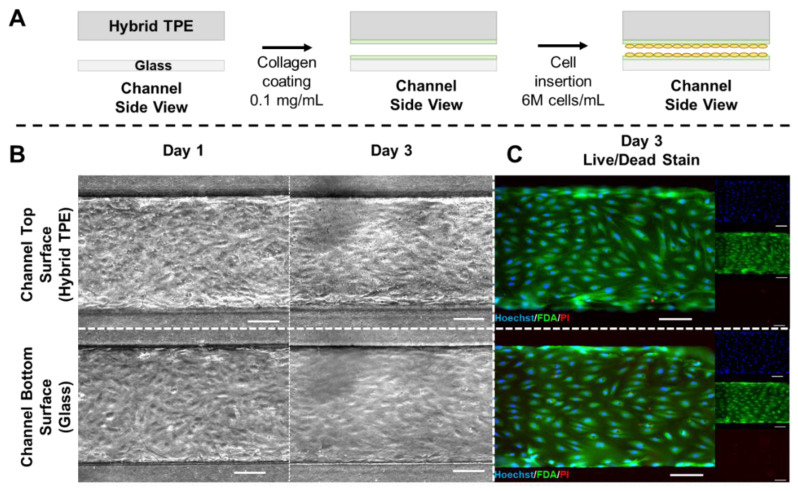
(**A**) Schematic depiction of cell culture experimental steps. The PC/TPE-hybrid–glass device is initially coated with type I collagen. Excess collagen is flushed out and followed by HUVEC seeding. The chip is temporarily flipped during cell seeding to ensure cell adhesion to both sides of the channel. (**B**) Micrographs acquired using phase contrast microscopy show cell attachment and progression of the cell culture from day 1 to day 3 for both substrates (left). (**C**) Fluorescence microscopy images from the live/dead staining (Hoechst/FDA/PI) on day 3 (left: composite image; right: individual color channels). Scale bars: 100 µm. *N* = 3.

In order to assess the suitability of the presented PC/TPE-hybrid material for microfluidic cell culture systems, HUVECs were seeded into the channels of PC/TPE-glass microfluidic devices and cultured for three days (Figure 4A). Bright-field microscopy at day 1 and day 3 revealed cell attachment to both glass and TPE surface as well as a physiological phenotype of the cells (Figure 4B), indicating that the material was not toxic to the cells. This observation was confirmed by live/dead staining performed on day 3, demonstrating almost no dead cells on both surfaces (Figure 4C). 

This observation is in line with previous reports from the literature where TPE-based systems were used for cell culture applications [25,26]. Overall, the combination of a lower absorption (compared to PDMS) and lack of cell toxicity demonstrate the potential of TPE systems for the development of cell-oriented applications as, e.g., in organ-on-chip technology.

## 4. Conclusions

In this work, we explored the use of TPE for rapid prototyping and facile fabrication of microfluidic devices, e.g., for cell culture and organ-on-chip applications. We introduced a simple and scalable method for patterning microchannels into TPE substrates. Combining TPE with a thin PC foil in a composite PC/TPE-hybrid material by simultaneous hot embossing and thermal bonding increases not only the ease of chip fabrication and handling but also the optical properties of the material. This composite material can be further bonded to different materials ranging from TPE to thermoplastics and glass. The successful bonding of TPE to thermoplastics could be achieved using a very simple thermal bonding process in a convection oven, without the need for pre-treatment, e.g., via oxygen plasma activation. Bonded devices showed to withstand 7.5 bar of applied pressure even after being submerged in liquid at cell culture conditions for a period of 7 days. This bonding strength is higher than previously reported for PDMS (to the same types of substrates) and also in a pressure range usually not applied in microfluidic systems, especially not for cell culture applications. Compared to other thermoplastics, TPE allows for simple and robust thermal fusion bonding and bypasses the need for solvents or adhesives, which can compromise cell-based applications. Additionally, we expanded the range of applications for TPE-based systems by enabling long-term stable bonding of TPE to glass via a novel approach based on bisamino silane functionalization. TPE/glass–hybrid devices combine the advantages of facile and diverse microfabrication approaches of TPE and the superior optical properties of glass, which is especially important for high-resolution imaging. To demonstrate the applicability of TPE-based devices for biotechnological applications, the absorption properties of TPE as well as the biocompatibility of the material was assessed. TPE absorbed significantly less small hydrophobic molecules in comparison to PDMS, demonstrating its viability as an alternative over PDMS for biosensing applications, amongst others. Moreover, cultivation of human endothelial cells in TPE-based devices did not show any indications of cytotoxicity. Overall, this work demonstrates the potential of TPE as an alternative to PDMS for microfluidic cell culture and organ-on-chip applications.

## Figures and Tables

**Table 1:** Maximum working pressures of PDMS- and TPE-based bonding to various substrates. Burst pressures from literature are converted to bar and rounded. TPE values (from this study) are average, maximum pressures of non-activated samples at day 0.

Substrate	PDMS [Bar]		TPE (This Study) [Bar]
PDMS	2.9–6.7	[41]	N/A
TPE	N/A		4.7 bar
Glass	1.7–5.1	[42]	>7.5 bar
PC	3.8	[43]	>7.5 bar
	4.1	[44]	
COC	>8	[45]	>7.5 bar
PS	4.5	[43,44]	>7.5 bar

## Data Availability

The data presented in this study are openly available in FigShare at 10.6084/m9.figshare.14610366.

## Supplementary Materials

The following are available online at https://www.mdpi.com/article/10.3390/mi12050575/s1, Figure S1: Epoxy mold fabrication, Figure S2: Bond strength assessment, Figure S3: PC/TPE-hybrid characterization, HTML-File: Analysis of rhodamine diffusion in microfluidic channels.

## References

[1] Jung W., Han J., Choi J.-W., Ahn C.H. (2015). Point-of-Care Testing (POCT) Diagnostic Systems Using Microfluidic Lab-on-a-Chip Technologies. Microelectron. Eng..

[2] Dittrich P.S., Manz A. (2006). Lab-on-a-Chip: Microfluidics in Drug Discovery. Nat. Rev. Drug Discov..

[3] Anderson S., Hadwen B., Brown C. (2021). Thin-Film-Transistor Digital Microfluidics for High Value in Vitro Diagnostics at the Point of Need. Lab Chip.

[4] Fernández-la-Villa A., Pozo-Ayuso D.F., Castaño-Álvarez M. (2019). Microfluidics and Electrochemistry: An Emerging Tandem for next-Generation Analytical Microsystems. Curr. Opin. Electrochem..

[5] Zhang B., Korolj A., Lai B.F.L., Radisic M. (2018). Advances in Organ-on-a-Chip Engineering. Nat. Rev. Mater..

[6] Schneider F., Draheim J., Kamberger R., Wallrabe U. (2009). Process and Material Properties of Polydimethylsiloxane (PDMS) for Optical MEMS. Sens. Actuators A Phys..

[7] Firpo G., Angeli E., Repetto L., Valbusa U. (2015). Permeability Thickness Dependence of Polydimethylsiloxane (PDMS) Membranes. J. Membr. Sci..

[8] Duffy D.C., McDonald J.C., Schueller O.J.A., Whitesides G.M. (1998). Rapid Prototyping of Microfluidic Systems in Poly(Dimethylsiloxane). Anal. Chem..

[9] Sip C.G., Folch A. (2014). Stable Chemical Bonding of Porous Membranes and Poly(Dimethylsiloxane) Devices for Long-Term Cell Culture. Biomicrofluidics.

[10] Lee K.S., Ram R.J. (2009). Plastic–PDMS Bonding for High Pressure Hydrolytically Stable Active Microfluidics. Lab Chip.

[11] Sunkara V., Park D.-K., Hwang H., Chantiwas R., Soper S.A., Cho Y.-K. (2011). Simple Room Temperature Bonding of Thermoplastics and Poly(Dimethylsiloxane). Lab Chip.

[12] Aran K., Sasso L.A., Kamdar N., Zahn J.D. (2010). Irreversible, Direct Bonding of Nanoporous Polymer Membranes to PDMS or Glass Microdevices. Lab Chip.

[13] Toepke M.W., Beebe D.J. (2006). PDMS Absorption of Small Molecules and Consequences in Microfluidic Applications. Lab Chip.

[14] Heo Y.S., Cabrera L.M., Song J.W., Futai N., Tung Y.-C., Smith G.D., Takayama S. (2007). Characterization and Resolution of Evaporation-Mediated Osmolality Shifts That Constrain Microfluidic Cell Culture in Poly(Dimethylsiloxane) Devices. Anal. Chem..

[15] Regehr K.J., Domenech M., Koepsel J.T., Carver K.C., Ellison-Zelski S.J., Murphy W.L., Schuler L.A., Alarid E.T., Beebe D.J. (2009). Biological Implications of Polydimethylsiloxane-Based Microfluidic Cell Culture. Lab Chip.

[16] Berthier E., Young E.W.K., Beebe D. (2012). Engineers Are from PDMS-Land, Biologists Are from Polystyrenia. Lab Chip.

[17] Volpatti L.R., Yetisen A.K. (2014). Commercialization of Microfluidic Devices. Trends Biotechnol..

[18] Tsao C.-W., DeVoe D.L. (2009). Bonding of Thermoplastic Polymer Microfluidics. Microfluid. Nanofluid..

[19] Troughton M.J. (2009). Solvent Welding. Handbook of Plastics Joining.

[20] Flachsbart B.R., Wong K., Iannacone J.M., Abante E.N., Vlach R.L., Rauchfuss P.A., Bohn P.W., Sweedler J.V., Shannon M.A. (2006). Design and Fabrication of a Multilayered Polymer Microfluidic Chip with Nanofluidic Interconnects via Adhesive Contact Printing. Lab Chip.

[21] Abeille F., Mittler F., Obeid P., Huet M., Kermarrec F., Dolega M.E., Navarro F., Pouteau P., Icard B., Gidrol X. (2014). Continuous Microcarrier-Based Cell Culture in a Benchtop Microfluidic Bioreactor. Lab Chip.

[22] Roy E., Galas J.-C., Veres T. (2011). Thermoplastic Elastomers for Microfluidics: Towards a High-Throughput Fabrication Method of Multilayered Microfluidic Devices. Lab Chip.

[23] Drobny J.G., Drobny J.G. (2014). 5—Styrenic Block Copolymers. Handbook of Thermoplastic Elastomers.

[24] Brassard D., Clime L., Li K., Geissler M., Miville-Godin C., Roy E., Veres T. (2011). 3D Thermoplastic Elastomer Microfluidic Devices for Biological Probe Immobilization. Lab Chip.

[25] Domansky K., Sliz J.D., Wen N., Hinojosa C., Thompson G., Fraser J.P., Hamkins-Indik T., Hamilton G.A., Levner D., Ingber D.E. (2017). SEBS Elastomers for Fabrication of Microfluidic Devices with Reduced Drug Absorption by Injection Molding and Extrusion. Microfluid. Nanofluid..

[26] Borysiak M.D., Bielawski K.S., Sniadecki N.J., Jenkel C.F., Vogt B.D., Posner J.D. (2013). Simple Replica Micromolding of Biocompatible Styrenic Elastomers. Lab Chip.

[27] Banerjee S., Burbine S., Kodihalli Shivaprakash N., Mead J. (2019). 3D-Printable PP/SEBS Thermoplastic Elastomeric Blends: Preparation and Properties. Polymers.

[28] Lee J., Choi K.-H., Yoo K. (2014). Innovative SU-8 Lithography Techniques and Their Applications. Micromachines.

[29] Huh D., Matthews B.D., Mammoto A., Montoya-Zavala M., Hsin H.Y., Ingber D.E. (2010). Reconstituting Organ-Level Lung Functions on a Chip. Science.

[30] Trietsch S.J., Israëls G.D., Joore J., Hankemeier T., Vulto P. (2013). Microfluidic Titer Plate for Stratified 3D Cell Culture. Lab Chip.

[31] Brás E.J.S., Fortes A.M., Esteves T., Chu V., Fernandes P., Conde J.P. (2020). Microfluidic Device for Multiplexed Detection of Fungal Infection Biomarkers in Grape Cultivars. Analyst.

[32] Zirath H., Rothbauer M., Spitz S., Bachmann B., Jordan C., Müller B., Ehgartner J., Priglinger E., Mühleder S., Redl H. (2018). Every Breath You Take: Non-Invasive Real-Time Oxygen Biosensing in Two- and Three-Dimensional Microfluidic Cell Models. Front. Physiol..

[33] Martin A., Teychené S., Camy S., Aubin J. (2016). Fast and Inexpensive Method for the Fabrication of Transparent Pressure-Resistant Microfluidic Chips. Microfluid. Nanofluid..

[34] Sticker D., Rothbauer M., Lechner S., Hehenberger M.-T., Ertl P. (2015). Multi-Layered, Membrane-Integrated Microfluidics Based on Replica Molding of a Thiol–Ene Epoxy Thermoset for Organ-on-a-Chip Applications. Lab Chip.

[35] Soares R.R.G., Varela J.C., Neogi U., Ciftci S., Ashokkumar M., Pinto I.F., Nilsson M., Madaboosi N., Russom A. (2020). Sub-Attomole Detection of HIV-1 Using Padlock Probes and Rolling Circle Amplification Combined with Microfluidic Affinity Chromatography. Biosens. Bioelectron..

[36] Rogal J., Binder C., Kromidas E., Roosz J., Probst C., Schneider S., Schenke-Layland K., Loskill P. (2020). WAT-on-a-Chip Integrating Human Mature White Adipocytes for Mechanistic Research and Pharmaceutical Applications. Sci. Rep..

[37] Sollier E., Murray C., Maoddi P., Di Carlo D. (2011). Rapid Prototyping Polymers for Microfluidic Devices and High Pressure Injections. Lab Chip.

[38] Terpilowski K., Rymuszka D. (2016). Surface Properties of Glass Plates Activated by Air, Oxygen, Nitrogen and Argon Plasma. Glass Phys. Chem..

[39] Soucy J.R., Bindas A.J., Koppes A.N., Koppes R.A. (2019). Instrumented Microphysiological Systems for Real-Time Measurement and Manipulation of Cellular Electrochemical Processes. iScience.

[40] Lachaux J., Alcaine C., Gómez-Escoda B., Perrault C.M., Duplan D.O., Wu P.-Y.J., Ochoa I., Fernandez L., Mercier O., Coudreuse D. (2017). Thermoplastic Elastomer with Advanced Hydrophilization and Bonding Performances for Rapid (30 s) and Easy Molding of Microfluidic Devices. Lab Chip.

[41] Eddings M.A., Johnson M.A., Gale B.K. (2008). Determining the Optimal PDMS–PDMS Bonding Technique for Microfluidic Devices. J. Micromech. Microeng..

[42] Bhattacharya S., Datta A., Berg J.M., Gangopadhyay S. (2005). Studies on Surface Wettability of Poly(Dimethyl) Siloxane (PDMS) and Glass under Oxygen-Plasma Treatment and Correlation with Bond Strength. J. Microelectromech. Syst..

[43] Sivakumar R., Trinh K.T.L., Lee N.Y. (2020). Heat and Pressure-Resistant Room Temperature Irreversible Sealing of Hybrid PDMS–Thermoplastic Microfluidic Devices via Carbon–Nitrogen Covalent Bonding and Its Application in a Continuous-Flow Polymerase Chain Reaction. RSC Adv..

[44] Sivakumar R., Lee N.Y. (2020). Chemically Robust Succinimide-Group-Assisted Irreversible Bonding of Poly(Dimethylsiloxane)–Thermoplastic Microfluidic Devices at Room Temperature. Analyst.

[45] Cortese B., Mowlem M.C., Morgan H. (2011). Characterisation of an Irreversible Bonding Process for COC–COC and COC–PDMS–COC Sandwich Structures and Application to Microvalves. Sens. Actuators B Chem..

[46] Beebe D.J., Mensing G.A., Walker G.M. (2002). Physics and Applications of Microfluidics in Biology. Annu. Rev. Biomed. Eng..

[47] Van Meer B.J., de Vries H., Firth K.S.A., van Weerd J., Tertoolen L.G.J., Karperien H.B.J., Jonkheijm P., Denning C., IJzerman A.P., Mummery C.L. (2017). Small Molecule Absorption by PDMS in the Context of Drug Response Bioassays. Biochem. Biophys. Res. Commun..

[48] Reese W.M., Burch P., Korpusik A.B., Liu S.E., Loskill P., Messersmith P.B., Healy K.E. (2020). Facile Macrocyclic Polyphenol Barrier Coatings for PDMS Microfluidic Devices. Adv. Funct. Mater..

[49] Moore T.A., Brodersen P., Young E.W.K. (2017). Multiple Myeloma Cell Drug Responses Differ in Thermoplastic vs. PDMS Microfluidic Devices. Anal. Chem..

