# Supplementary material for: Facile Patterning of Thermoplastic Elastomers and Robust Bonding to Glass and Thermoplastics for Microfluidic Cell Culture and Organ-on-Chip
Source: Micromachines (Basel). 2021 May 18;12(5):575. doi: 10.3390/mi12050575 (PMC8158514; doi:10.3390/mi12050575)
Supplement: Supplementary file 1 [file micromachines-12-00575-s001.zip › micromachines-1211883 - Supplementary_Information for conversion.pdf]

micromachines

MDPI

Supplementary Information: Facile Patterning of Thermoplastic Elastomers and Robust Bonding to Glass and Thermoplastics for Microfluidic Cell Culture and Organ-On-Chip

Epoxy mold fabrication

To fabricate the epoxy master mold used for the hot embossing of the PC/TPE-hybrid material, it is necessary to remove all air enclosed in the epoxy mixture. In order to perform this step while maintaining a flat epoxy, a custom aluminum tool was designed and fabricated by an external supplier (CNCTeile24). This tool allows for the placement of the PDMS negative mold and the application of a negative pressure through the PDMS layer to ensure full compression of the epoxy mixture against the PDMS features (Figure S1).

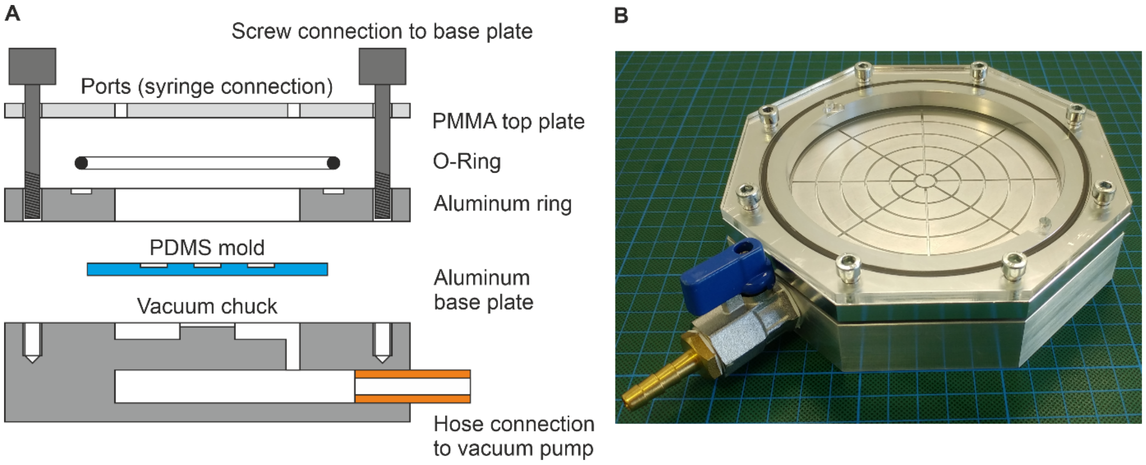

Figure S1. (**A**) Schematic side view and (**B**) picture of the custom built aluminum tool for the casting of the epoxy master mold.

Bond strength assessment

To determine the robustness of the bonding methods used, the microfluidic chips were submerged in a water basin as the applied pressure was increased (Figure S2). This allowed early detection of air bubbles resulting from structure failure caused by delamination.

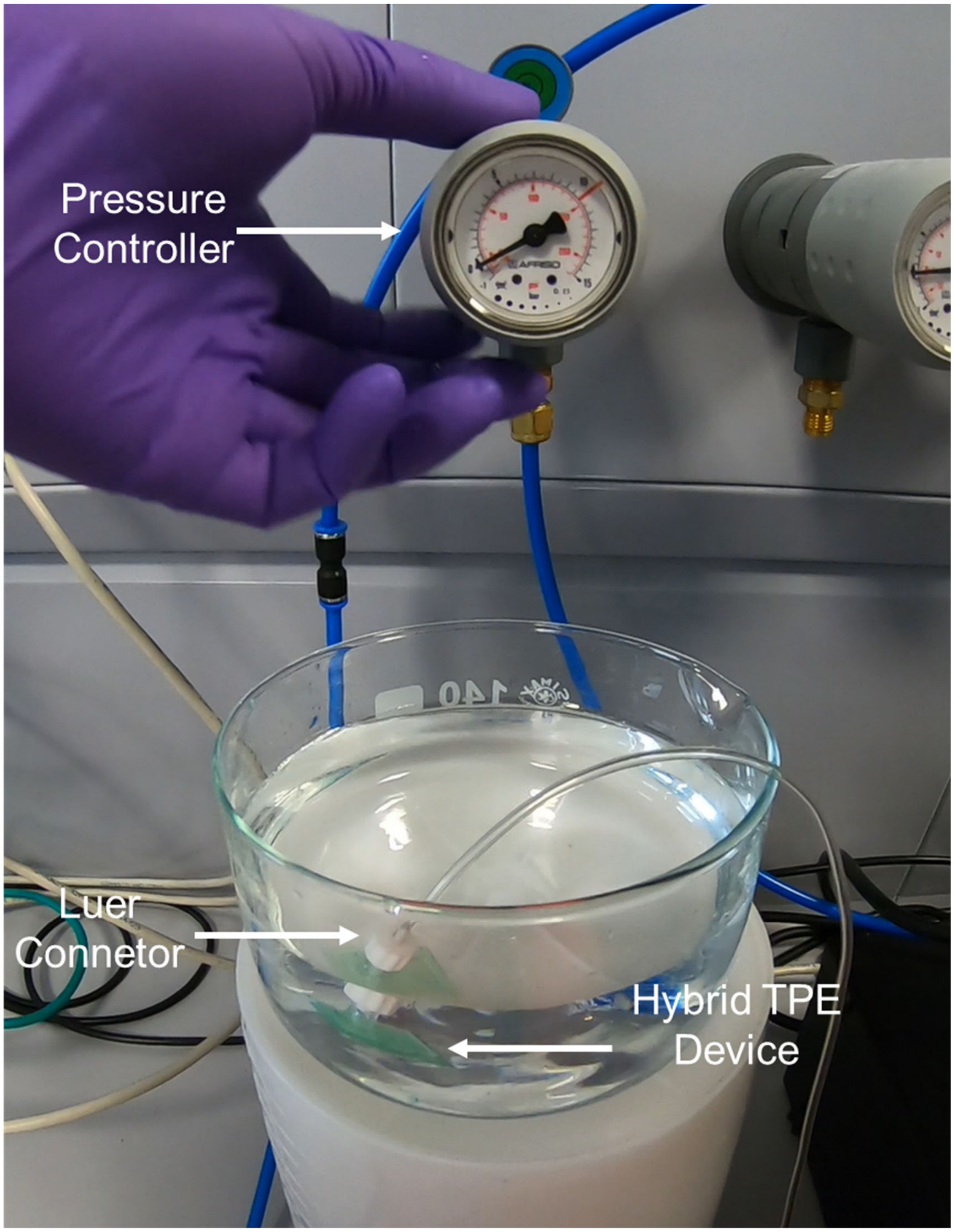

Figure S2. High pressure setup used for assessing bonding strength. The pressure controller was connected to the PC/TPE-hybrid chip using PTFE tubing and a luer connector.

PC/TPE-hybrid characterization

The PC/TPE-hybrid is fabricated using a 750 μm thick sheet of TPE and a 500 μm foil of PC. During the embossing step, some of the material is ejected towards the sides of the mold resulting in a composite with a final thickness of 1100 μm, which retains properties of both the original materials (Figure S3).

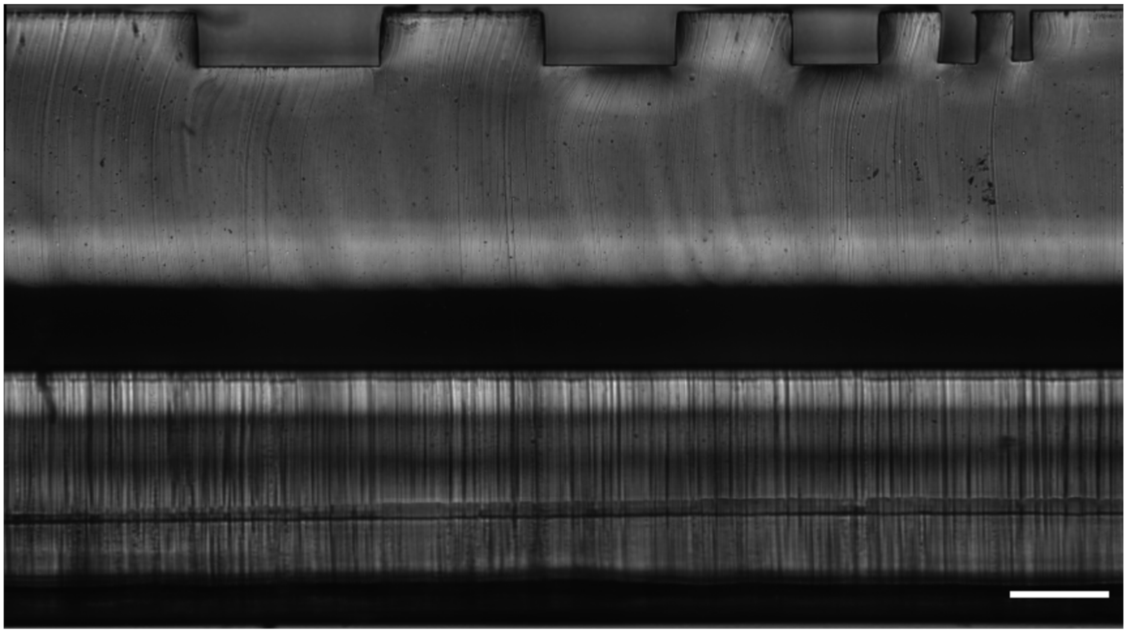

Figure S3. Side view of the composite PC/TPE-hybrid material. Scale bar represents 200 μm.

Analysis of rhodamine diffusion in microfluidic channels

The source code of the custom python script for the analysis of rhodamine diffusion in the microfluidic channels is accessible online (https://github.com/loslab/rhodabs) and in the supplementary HTML file.
