# Supplementary material for: Facile Patterning of Thermoplastic Elastomers and Robust Bonding to Glass and Thermoplastics for Microfluidic Cell Culture and Organ-on-Chip
Source: Micromachines (Basel). 2021 May 18;12(5):575. doi: 10.3390/mi12050575 (PMC8158514; doi:10.3390/mi12050575)
Supplement: Supplementary file 1 [file micromachines-12-00575-s001.zip › Supplementary_Information_source_code.html]

extract\_profiles-SI


# Analysis of rhodamine diffusion in microfluidic channels¶

- conditions: 3 different rhodamines (Rh 101, Rh 6G, Rh B) injected into chips fabricated from 2 materials (PDMS, TPE)
- fluorescence images are acquired after injection (d0), on days d1, d2, d3 and after washout on d3 (d3\_wash)
- images are rotated and centered
- profiles are then extracted and averaged & normalized to compare diffusion behaviour

## Package imports¶

In [1]:

```
import tifffile
import pathlib
from scipy import ndimage, stats
import numpy as np
import matplotlib.pyplot as plt
from itertools import cycle
import pandas as pd
from IPython.display import Markdown, display
```

## Specify constants and inputfiles¶

recorded with 10x objective, scale = 1.5374 px/micron (= 0.6504 micron/px) at 2048 x 2048 resolution

In [2]:

```
resolution_ppm = 1.5374
imsize_px = 2048
```

In [3]:

```
timepoints = ["d0", "d1", "d2", "d3", "d3_wash"]
inputfolders =  {timepoint: pathlib.Path(timepoint) for timepoint in timepoints}
# select samples with *002 suffix corresponding to images taken in channel center
inputfiles = {key: list(folder.glob('*002.tif')) for key, folder in inputfolders.items()}
```

## Auxiliary functions¶

### Edge detection and image rotation¶

In [4]:

```
def rotate_img(path):
    """
        rotates image by detecting edge in brightfield
        input: path to multichannel .tif, channel 0: BF, channel 1: fluo
        output: saves rotated BF for visual control and rotated fluo for further analysis
    """
    img = tifffile.imread(str(path)) # load image
    
    # detect edge on brightfield
    bf = img[0]
    blur = ndimage.gaussian_filter(bf, sigma=10)
    
    blur_crop = blur[:,0:int(imsize_px/2)] # select left half of image to detect edge, works as image is roughly centered
    
    grad = np.gradient(blur_crop, axis= 1)    # take gradient along x-axis
    grad[grad>10000]=0    # filter unreasonable high values
    grad[grad<np.nanmean(grad)] = 0
    
    maxline = np.argmax(grad, axis=1) # get index of max. along x-axis

    idx = np.arange(maxline.shape[0])
    xmean = np.mean(maxline)
    xstd = np.std(maxline)
    idx_filtx = idx[(maxline < xmean+xstd) & (maxline > xmean - xstd)]    # filter positions where highest slope is at different xposition(blobs)
    ymean = idx_filtx.mean()
    ystd = idx_filtx.std()
    idx_filtxy = idx_filtx[(idx_filtx < ymean + ystd) & (idx_filtx > ymean - ystd)] # filter positions where highest slope is at different yposition

    # fit line to edge defined by filtered region and extract slope
    slope, intercept, *fitstats = stats.linregress(idx_filtxy, maxline[idx_filtxy])
    """
    # alternative approach: threshold and select biggest region
    #grad = grad>np.nanmean(grad) # threshold by mean
    regions, Nregions = ndimage.label(grad)
    regionsize = np.bincount(region.ravel())
    biggestregion = regionsize[1:].argmax() + 1
    edgeregion = (regions == biggestregion)
    """    
    
    # rotate brightfield and save to allow for visual control
    bf_save = ndimage.rotate(bf,-slope*180/np.pi, reshape = False)
    bf_rotatefolder = path.parent/"BF_rotated"
    bf_rotatefolder.mkdir(parents=True, exist_ok=True)
    tifffile.imsave(str(bf_rotatefolder/path.name), bf_save)

    # rotate fluo-images by same angle and save
    fluo = img[1]
    fluo = ndimage.rotate(fluo,-slope*180/np.pi, reshape = False)
    fluo_rotatefolder = path.parent/"Fluo_rotated"
    fluo_rotatefolder.mkdir(parents=True, exist_ok=True)
    tifffile.imsave(str(fluo_rotatefolder/path.name), fluo)
```

demonstration of tilt extraction on one image

In [5]:

```
testfile = inputfiles["d0"][10]
img = tifffile.imread(str(testfile)) # load image
    
# detect edge on brightfield
bf = img[0]
blur = ndimage.gaussian_filter(bf, sigma=10)
    
blur_crop = blur[:,0:int(imsize_px/2)] # select left half of image to detect edge, works as image is roughly centered
grad = np.gradient(blur_crop, axis= 1)    # take gradient along x-axis
grad[grad>10000]=0    # filter unreasonable high values
grad[grad<np.nanmean(grad)] = 0

maxline = np.argmax(grad, axis=1) # get index of max. along x-axis

idx = np.arange(maxline.shape[0])
xmean = np.mean(maxline)
xstd = np.std(maxline)
idx_filtx = idx[(maxline < xmean+xstd) & (maxline > xmean - xstd)]    # filter positions where highest slope is at different xposition(blobs)
ymean = idx_filtx.mean()
ystd = idx_filtx.std()
idx_filtxy = idx_filtx[(idx_filtx < ymean + ystd) & (idx_filtx > ymean - ystd)] # filter positions where highest slope is at different yposition

# fit line to edge defined by filtered region and extract slope
slope, intercept, *fitstats = stats.linregress(idx_filtxy, maxline[idx_filtxy])

fig, ax = plt.subplots(1,3, figsize = (10,4), dpi=150)
ax[0].imshow(grad, cmap='gray', origin = 'lower')
ax[0].set_ylabel('y [px]')
ax[0].set_xlabel('x [px]')
ax[0].set_title('gradient in x of image')

ax[1].plot(maxline[idx], idx, ls='none', marker = 'o')
ax[1].set_ylabel('y [px]')
ax[1].set_xlabel('x [px]')
ax[1].set_title('edge from linewise max')

ax[2].plot(maxline[idx_filtxy], idx_filtxy, ls='none', marker = 'o', label = 'filtered edge')
ax[2].plot(idx_filtxy*slope+intercept, idx_filtxy, ls='-', c= 'r', label = 'linear fit')
ax[2].set_ylabel('filtered y [px]')
ax[2].set_xlabel('filtered x [px]')
ax[2].set_title('filtering and tilt extraction')
ax[2].legend()
ax[2].text(0.5,0.75,"tilt: {:.2f}°".format(slope*180/np.pi), transform=ax[2].transAxes)
fig.tight_layout()
```

### Profile centering + averaging¶

In [6]:

```
def get_profile_center(path):
    """
        extracts profile along channel
        centers fluo-image and saves centered version, cropped to 700 px on each side around center
        input: path to fluo-image
        output: centered profile, saves centered fluo-image
    """
    
    fluo = tifffile.imread(str(path)) # load image

    px_crop = 150 # rotated image has black boarder -> crop each image
    fluo_cropped = fluo[0+px_crop:imsize_px-px_crop,0+px_crop:imsize_px-px_crop]
    profile = fluo_cropped.mean(axis=0) # get mean along y-axis
    
    # detect both edges by identifying mean intensity
    max_10 = np.percentile(profile,90)
    min_10 = np.percentile(profile,10)
    mid_I = (max_10+min_10)/2
    # find indices where I ~ mid_I
    idx = ((np.abs(profile - mid_I))<0.05*max_10).nonzero()
    x_low = np.min(idx)
    x_up = np.max(idx)
    center = int((x_up+x_low)/2)
    
    # create centerd x-values
    arrlen = profile.shape[0]
    x_centered = np.arange(arrlen) - center
    centered_profile = np.array([x_centered, profile])    
    
    # center image and save
    shift = arrlen/2-center
    fluo_center = ndimage.shift(fluo_cropped,(0,shift))

    imgcent = int(arrlen/2)
    center_cropped = fluo_center[imgcent-700:imgcent+700,imgcent-700:imgcent+700]
    centerfolder = path.parent.parent/"Fluo_centered"
    centerfolder.mkdir(parents=True, exist_ok=True)
    tifffile.imsave(str(centerfolder/path.name), center_cropped, imagej=True, resolution=(resolution_ppm, resolution_ppm), metadata={'unit': 'um'}, photometric = 'minisblack')      
    return centered_profile
```

In [7]:

```
def get_mean_profile(profiles_centered, profilenames):
    """
        extract mean profile of various samples of same condition
        input:  dict of centered profiles & profilenames (=keys of dict)
                to be used for calculation of mean
    """
    
    serieslist = []
    for name in profilenames:
        profile = profiles_centered[name]
        newentry = pd.Series(profile[1],index=profile[0], name=name)
        serieslist.append(newentry)
        # following https://stackoverflow.com/questions/41821539/calculate-average-of-y-values-with-different-x-values
    
    mean_I = pd.concat(serieslist, axis=1).mean(axis=1)
    mean_profile = np.array([mean_I.index, mean_I.values])
    return mean_profile
```

## Processing of images¶

### Rotate images¶

In [8]:

```
for folder, files in inputfiles.items():
    for file in files:
        rotate_img(file)
```

visual check of brightfield images after rotation in folder BF\_rotated confirms correct rotation

### Center fluorescence images + get profile¶

In [9]:

```
fluo_rotated = {day: list((folder/'Fluo_rotated').glob('*002.tif')) for day, folder in inputfolders.items()}
profiles_centered = {}

for day, files in fluo_rotated.items():
    
    dayprofiles = {}
    for file in files:
        profile = get_profile_center(file)
        sample = file.stem
        dayprofiles[sample] = profile
        
    profiles_centered[day] = dayprofiles
```

### Get mean profile for each day + material/ Rhodamine condition¶

In [10]:

```
meansamples = {'PDMS_Rh101': ['PDMS-101-1-002','PDMS-101-2-002', 'PDMS-101-3-002'],
            'PDMS_Rh6G': ['PDMS-6G-1-002', 'PDMS-6G-2-002', 'PDMS-6G-3-002'],
            'PDMS_RhB': ['PDMS-B-1-002','PDMS-B-2-002', 'PDMS-B-3-002'],
            'TPE_Rh101':  ['TPE-101-1-002','TPE-101-2-002', 'TPE-101-4-002'],
            'TPE_Rh6G':  ['TPE-6G-1-002','TPE-6G-2-002', 'TPE-6G-4-002'],
            'TPE_RhB':  ['TPE-B-1-002','TPE-B-2-002', 'TPE-B-3-002'],
           }

# exclude PDMS-6G-2
meansamples_excl = {'PDMS_Rh101': ['PDMS-101-1-002','PDMS-101-2-002', 'PDMS-101-3-002'],
            'PDMS_Rh6G': ['PDMS-6G-1-002', 'PDMS-6G-3-002'],
            'PDMS_RhB': ['PDMS-B-1-002','PDMS-B-2-002', 'PDMS-B-3-002'],
            'TPE_Rh101':  ['TPE-101-1-002','TPE-101-2-002', 'TPE-101-4-002'],
            'TPE_Rh6G':  ['TPE-6G-1-002','TPE-6G-2-002', 'TPE-6G-4-002'],
            'TPE_RhB':  ['TPE-B-1-002','TPE-B-2-002', 'TPE-B-3-002'],
           }

meanprofiles = {}
for day in ["d0", "d1","d2"]:
    meanprofiles[day] = {condition: get_mean_profile(profiles_centered[day], profilenames) for condition, profilenames in meansamples.items() }
for day in ["d3", "d3_wash"]:
    meanprofiles[day] = {condition: get_mean_profile(profiles_centered[day], profilenames) for condition, profilenames in meansamples_excl.items() }    
    
# meanprofiles is dict with structure: [day][condition][profile(2D-array)]
```

### Determine mean in central region on d0 to normalize intensities¶

In [11]:

```
norms = {}
center = int(meanprofiles['d0']['PDMS_RhB'][1].shape[0]/2)
meanslice = np.s_[1,center-100:center+100]
d0_profiles = meanprofiles['d0']
norms['RhB']=(d0_profiles['PDMS_RhB'][meanslice].mean() + d0_profiles['TPE_RhB'][meanslice].mean() )/2
norms['Rh6G']=(d0_profiles['PDMS_Rh6G'][meanslice].mean() + d0_profiles['TPE_Rh6G'][meanslice].mean() )/2
norms['Rh101']=(d0_profiles['PDMS_Rh101'][meanslice].mean() + d0_profiles['TPE_Rh101'][meanslice].mean() )/2
```

## Plotting of mean profiles¶

In [12]:

```
import matplotlib.ticker
matplotlib.rcParams['svg.fonttype'] = 'none' # text will be exported as text in svg
```

In [13]:

```
display(Markdown("## Output"))
Rhodamines = ['Rh101', 'Rh6G', 'RhB']

for Rhodamine in Rhodamines:
    
    norm = norms[Rhodamine]/100

    fig, axs = plt.subplots(1,2, figsize = (8,2.5), sharex= 'col',sharey='row',dpi=100)
    fig.suptitle(Rhodamine)

    material = 'PDMS'
    profilename = material+'_'+Rhodamine
    ax = axs[0]
    ax.set_ylabel('Intensity [a.u.]', fontname='Arial')
    
    for day in timepoints:
        ax.plot(meanprofiles[day][profilename][0]/resolution_ppm,meanprofiles[day][profilename][1]/norm, label=day)
    
    material = 'TPE'
    profilename = material+'_'+Rhodamine

    ax = axs[1]

    for day in timepoints:
        ax.plot(meanprofiles[day][profilename][0]/resolution_ppm, meanprofiles[day][profilename][1]/norm, label=day)

    ax.yaxis.set_tick_params(labelleft=True)
    ax.yaxis.tick_right()
    
    for ax in axs:
        ax.set_xlim(-700/resolution_ppm,700/resolution_ppm)
        ax.set_ylim(0)
        ax.set_xlabel('Distance to channel center [µm]', fontname='Arial')
        ax.tick_params(axis='both', which='both', direction='in', top=True, right=True, left=True)

        ax.xaxis.set_minor_locator(matplotlib.ticker.AutoMinorLocator(n=2)) # places 1 minor tick between each major tick
        ax.yaxis.set_minor_locator(matplotlib.ticker.AutoMinorLocator(n=2))
        
    lines = axs[1].get_lines()
    axs[1].legend(lines,["0 h", "24 h", "48 h", "72 h", "72 h washout"], loc="center left", bbox_to_anchor=(0.8,0.5))

    fig.savefig(Rhodamine+'_combined_mean.svg',dpi=300)
    fig.savefig(Rhodamine+'_combined_mean.png',dpi=300)
```

## Output¶
